# Real-time Ultrasound-Guided Manual Testicular Detorsion: A Case Report

**DOI:** 10.5811/cpcem2022.6.57256

**Published:** 2022-08-08

**Authors:** Wilson T. Smith, Stephanie Midgley, Tobias Kummer

**Affiliations:** * Vassar Brothers Medical Center, Department of Emergency Medicine, Poughkeepsie, New York; † Mayo Clinic, Department of Emergency Medicine, Rochester, Minnesota

**Keywords:** testicular torsion, detorsion, ultrasound, color Doppler, case report

## Abstract

**Introduction:**

Acute testicular torsion is a surgical emergency due to acute testicular ischemia. Manual testicular detorsion is a testis-saving, bedside therapeutic when performed correctly and in a timely fashion. This procedure is most commonly performed blindly with pain relief as the endpoint for detorsion. However, up to one-third of patients continued to show signs of residual torsion in the operating room even using pain relief as the stopping point for the procedure.

**Case Report:**

We present a case demonstrating the utility of color Doppler ultrasound to confirm complete manual detorsion in a 14-year-old male with acute testicular torsion. The patient underwent 360-degree detorsion and had relief of pain; however, color Doppler demonstrated incomplete return of flow to the testis. After an additional 180-degree turn was made, color Doppler demonstrated complete return of normal vascular flow to the torsed testis.

**Conclusion:**

When it comes to testicular viability, timely restoration of blood flow to the testicle is of utmost importance. Manual detorsion is a non-invasive intervention that can be quickly and effectively performed at the bedside. Moreover, using color Doppler ultrasound guidance can ensure that physicians detorse in the proper direction and to completion, by providing instant visualization of restorative flow and ensuring reperfusion of the testis while awaiting definitive surgical management.

## INTRODUCTION

The estimated yearly incidence of testicular torsion in males under the age of 18 presenting to the emergency department (ED) is 3.8 per 100,000.^1^ Testicular torsion is a urological emergency due to the negative effect of prolonged ischemia on testicular function and viability. For every 10-minute delay in the ED, the chance of having a viable testis decreases 4.8%.^4^ Within four hours of symptom onset, the torsed testis begins to atrophy, resulting in lower sperm counts and potential infertility. Moreover, long-term semen analysis may only be normal in up to 50% of these patients.^3^ As the time to detorsion increases, the viability of the testis decreases. Due to current coronavirus 2019 pandemic challenges, prompt surgical treatment may be delayed, making manual detorsion even more critical. Manual detorsion in the ED is a manipulative technique that can restore blood flow and preserve the long-term viability of the testis and provide dramatic pain relief.

Manual testicular detorsion is a bedside procedure taught to emergency care practitioners and performed blindly. Conventional emergency medicine teaching dictates that torsions occur in the lateral-to-medial direction and, therefore, detorsion should be performed in the medial-to-lateral or “open-book” fashion until pain relief.^5^ Although this is true for most cases, Sessions et al found that as many as 30% of testicular torsions require lateral-to-medial manipulation to successfully detorse.^6^ Additionally, residual torsion was discovered in the operating room (OR) in as many as one-third of cases in which a patient had undergone blind manual detorsion using pain resolution as the determinant to stop detorsing.^6^

With as little as 180 degrees of torsion causing testicular infarct,^6^ ensuring complete detorsion while awaiting surgical correction is of the utmost importance. Color Doppler ultrasound (CDU), which is used to confirm the diagnosis of testicular torsion, has also been reported to guide detorsion.^9^,^10^ By using CDU to visualize the return of vascular flow, it can demonstrate that the direction of detorsion is correct and confirm complete detorsion while awaiting definite surgical intervention. This technique can be used in real time by assessing vascular flow after each rotation or by assessing flow at the end of the procedure. There have been no studies comparing blind manual detorsion vs CDU guided, but futures studies may determine that CDU-guided detorsion is a superior method to ensuring complete detorsion.

## CASE REPORT

**Image 1:**
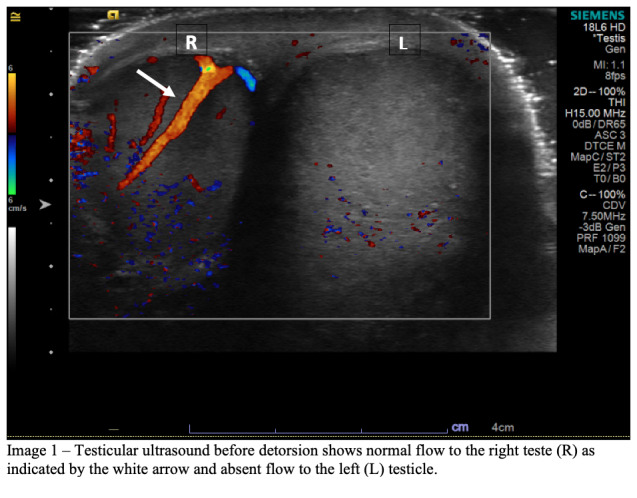
Testicular ultrasound before detorsion shows normal flow to the right testicle (R) as indicated by the white arrow and absent flow to the left (L) testicle.

A 14-year-old male presented to the ED with vomiting and sudden onset of left testicular pain, waking him up from sleep approximately two hours prior. The patient denied experiencing trauma, fever, chills, diarrhea, or difficulty urinating. Physical examination revealed a high-riding left testicle and absent left-sided cremasteric reflex without erythema or swelling. Scrotal ultrasound was performed in the radiology ultrasound suite using both color Doppler and spectral Doppler. Both testes had normal echotexture and appeared to be of the same size. Arterial and venous waveforms were identified in the right testis but were absent on the left (Image 1).

**Image 2:**
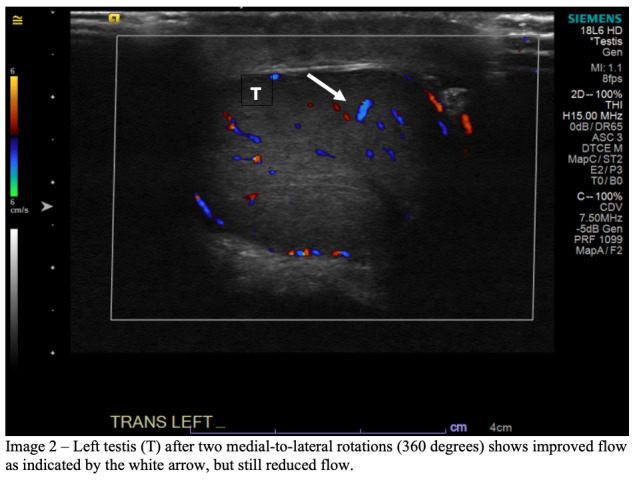
Left testicle (T) after two medial-to-lateral rotations (360 degrees) shows improved flow as indicated by the white arrow, but still reduced flow.

**Image 3:**
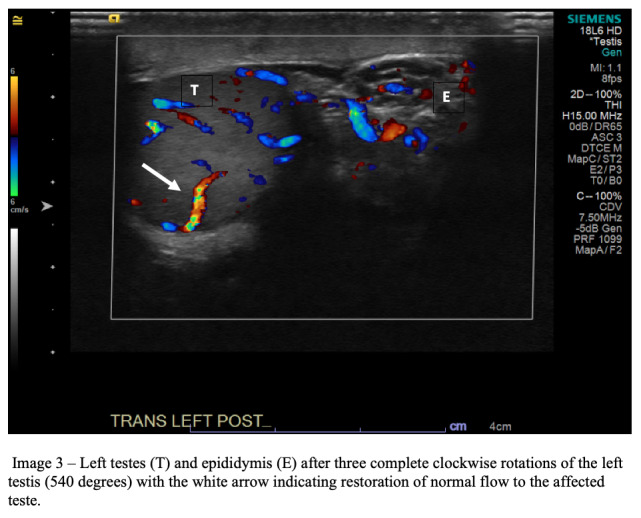
Left testicle (T) and epididymis (E) after three complete clockwise rotations of the left testicle (540 degrees) with the white arrow indicating restoration of normal flow to the affected teste.

In the ultrasound suite, with the aid of the ultrasound technician, the emergency physician manually detorsed the left testicle by turning it from medial to lateral and re-evaluated for return of flow with CDU after each 180-degree turn. After the first 180-degree rotation, the patient’s pain did not begin to decrease, bringing into question torsion directionality. Color Doppler ultrasound demonstrated a small increase in vascular flow to the left testis, suggesting the correct detorsion direction but the need for further rotations. An additional 180-degree rotation was performed, resulting in a marked decrease in pain; however, repeat CDU showed no improvement in flow. Given the continued paucity of vascular flow to the left testis when compared to the right (Image 2) there was concern for only partial detorsion; thus, a third 180-degree rotation (540° total) was performed, which brought additional pain relief, and CDU showed return of normal vascular flow to the left testis (Image 3).

**CPC-EM Capsule**

What do we already know about this clinical entity?

*Testicular torsion is a surgical emergency and manual testicular detorsion, when performed correctly, is a testis-saving bedside therapeutic*.

What makes this presentation of disease reportable?

*Color Doppler ultrasound detorsion guidance was performed with the aid of an ultrasound technician to confirm complete testicular detorsion with reperfusion*.

What is the major learning point?

*Color Doppler ultrasound guidance can be used to guide manual testicular detorsion for correct direction and confirm complete detorsion*.

How might this improve emergency medicine practice?

*Color Doppler ultrasound guided testicular detorsion can be performed both as a point-of-care ultrasound procedure or performed with an ultrasound technician*.

The on-call urologist then brought the patient to the OR for bilateral testicular orchiopexy. During surgery, the left testicle was delivered from the left hemiscrotum and was noted to be completely detorsed, pink, and viable. There were no surgical complications, and the patient was discharged that day.

## DISCUSSION

Testicular torsion accounts for up to 25% of pediatric cases of acute scrotal pain.^7^ Color Doppler ultrasound is useful in both diagnosing and treating testicular torsion. In a comprehensive literature review, Chen and Esler found that CDU had a diagnostic sensitivity of 91.9% and specificity of 98.9% when confirmed with surgical findings.^7^ Once the diagnosis of torsion has been made, and manual detorsion is attempted, the direction and degree of rotations must be determined. However, there is a lack of studies assessing the use of real-time ultrasound for guidance during detorsion attempts.

Conventional teaching dictates that to detorse a testis, it should be manually rotated in a medial-to-lateral direction.^5^ However, data of 200 testicular torsions confirmed by surgical findings collected over 20 years showed that only two-thirds of the time was the testis torsed medially.^6^ One study by Hosokawa showed that the directionality of testicular torsion can be better identified with CDU. In that study, CDU correctly predicted directionality 78.6% of the time.^8^ The key finding was the so-called “whirlpool sign,” a spiral twist of the spermatic cord to the extent that its patency is decreased or completely occluded.^11^ Once the direction of detorsion has been determined, the next step is to determine the number of rotations to be made when attempting manual testicular detorsion. Rotations can be performed in 180-degree increments using CDU to assess for the restoration of vascular flow after each turn. Although pain resolution has been associated with testicular reperfusion, residual torsion was discovered in the OR in as many as one-third of cases in which a patient had undergone manual detorsion without the use of ultrasound.^6^ With as little as 180 degrees of torsion causing testicular infarct,^6^ using real-time CDU guidance becomes even more prudent to ensure complete detorsion while waiting for definitive surgical treatment.

## CONCLUSION

When it comes to testicular viability, timely restoration of blood flow to the testicle is of utmost importance. Manual detorsion is a non-invasive intervention that can be quickly and effectively performed at the bedside. Moreover, using CDU guidance can ensure that physicians detorse in the proper direction and to completion, by providing instant visualization of restorative flow and ensuring reperfusion of the testis while awaiting definitive surgical management. Prior case reports demonstrate CDU guidance as a viable point-of-care US (POCUS) procedure.^9^,^13^ However, the cases were performed by more experienced ultrasound-fellowship trained emergency physicians. Testicular ultrasound is an advanced clinical ultrasound application, requiring additional training beyond residency. Many emergency physicians have not had additional ultrasound training, which limits their ability to perform POCUS-guided detorsion. This case demonstrates that CDU detorsion guidance can be successfully performed with the aid of an ultrasound technician. This allows emergency physicians without additional POCUS training the benefit of using this technique for guiding their manual detorsion.
